# Exercise as a Mean to Control Low-Grade Systemic Inflammation

**DOI:** 10.1155/2008/109502

**Published:** 2009-01-11

**Authors:** Neha Mathur, Bente Klarlund Pedersen

**Affiliations:** The Centre of Inflammation and Metabolism (CIM), Department of Infectious Diseases and CMRC, Rigshospitalet, Faculty of Health Sciences, University of Copenhagen, Blegdamsvej 9, 2100 Copenhagen, Denmark

## Abstract

Chronic noncommunicable diseases (CNCDs), which include cardiovascular disease, some cancers, for example, colon cancer, breast cancer, and type 2 diabetes, are reaching epidemic proportions worldwide. It has now become clear that low-grade chronic inflammation is a key player in the pathogenesis of most CNCDs. Given that regular exercise offers protection against all causes of mortality, primarily by protection against atherosclerosis and insulin resistance, we suggest that exercise may exert some of its beneficial health effects by inducing anti-inflammatory actions. Recently, IL-6 was introduced as the first myokine, defined as a cytokine, which is produced and released by contracting skeletal muscle fibres, exerting its effects in other organs of the body. We suggest that skeletal muscle is an endocrine organ and that myokines may be involved in mediating the beneficial effects against CNCDs associated with low-grade inflammation.

## 1. INTRODUCTION

Chronic noncommunicable diseases (CNCDs) which include cardiovascular conditions (mainly heart diseases and stroke), some cancers, chronic respiratory conditions, and type 2 diabetes, affect people of all nationalities and classes and are reaching epidemic proportions worldwide [1–4].

CNCDs cause the greatest global share of death and disability, accounting for around 60% of all deaths worldwide. Approximately, 80% of chronic-disease deaths occur in low- and middle-income countries and account for 44% of premature deaths worldwide. It is estimated that the number of deaths from these diseases is double the number of deaths that result from a combination of infectious diseases (including HIV/AIDS, tuberculosis, and malaria), maternal and perinatal conditions, and nutritional deficiencies [1].

From a historical perspective, inflammation has been considered as the natural host response to an acute infectious episode, whereas chronic inflammation has been considered a sign of chronic infection. It has now become clear that low-grade chronic inflammation is a key player in the pathogenesis of most NCDCs. There has been an increasing appreciation of the role of inflammation both in the pathogenesis of atherosclerosis [5, 6] and as a key factor in insulin resistance [7]. Low-grade chronic inflammation is characterized by increased systemic levels of some cytokines and C-reactive protein (CRP) and a number of studies have confirmed an association between low-grade systemic inflammation on one hand and atherosclerosis and type 2 diabetes on the other [8].

Physical inactivity has been identified as a stronger predictor of these chronic diseases than risk factors such as hypertension, hyperlipidemia, diabetes, and obesity for all-cause mortality [9]. Moreover, regular physical activity appears to protect against premature death independent of obesity [10, 11]. 

Regular physical activity offers protection against, and may be useful as a treatment for a wide variety of, chronic diseases associated with low-grade inflammation [12]. The protective effects of regular exercise against diseases such as cardiovascular disease, type 2 diabetes, colon cancer, and breast cancer have been reviewed extensively [13–16].

Recent findings demonstrate that physical activity induces an increase in the systemic levels of a number of cytokines with anti-inflammatory properties [8, 17] and skeletal muscle has recently been identified as an endocrine organ, which produces and releases cytokines (also called myokines) [18, 19].

The discovery of contracting muscle as a cytokine-producing organ opens a new paradigm; skeletal muscle is an endocrine organ that, by contraction, stimulates the production and release of myokines, which may influence metabolism and modify cytokine production in tissue and organs.

## 2. CHRONIC LOW-GRADE SYSTEMIC INFLAMMATION AND ITS CONSEQUENCES

In response to an acute infection or trauma, the cytokines and cytokines inhibitors will increase [20]. The initial cytokines as they appear in the circulation in relation to an acute infection consist of the following: TNF-*α*, IL-1*β*, IL-6, IL-1 receptor antagonist (IL-1ra), and soluble TNF-*α*-receptors (sTNF-R), and IL-10. The systemic response known as the acute-phase response includes the production of a large number of hepatocyte-derived acute phase proteins, such as C-reactive protein (CRP) that is known to be a sensitive marker of systemic inflammation. The response can be mimicked by the injection of the cytokines TNF-*α*, IL-1*β*, and IL-6 into laboratory animals or humans [8, 21]. Chronic low-grade systemic inflammation has been characterized by a 2- to 3-fold elevation in the systemic concentrations of proinflammatory and anti-inflammatory cytokines, natural occurring cytokine antagonists, and the acute phase reactant C-reactive protein (CRP) [13, 22]. In the latter case, the stimuli for the cytokine production are not known, but it is assumed that the origin of TNF in chronic low-grade systemic inflammation is mainly the adipose tissue [23–26].

Type 2 diabetes, obesity, and cardiovascular disease are related to a state of low-grade systemic inflammation [7, 27–29]. Despite the fact that the changes in acute-phase reactants are much smaller than those in acute infections, the chronicity of low-grade inflammation is strongly associated with increasing age, lifestyle factors such as smoking and obesity, together with increased risk of cardiovascular disease and type 2 diabetes [22, 25, 30]. Plasma concentrations of IL-6 [31] and TNF-*α* have been shown to predict the risk of myocardial infarction in several studies [32], and CRP has emerged as a particularly stronger independent risk factor for cardiovascular disease than the low-density lipoprotein cholesterol level [13, 33, 34]. 

## 3. THE DIRECT METABOLIC ROLES OF TNF

Growing evidence suggests that TNF-*α* plays a direct role in metabolic syndrome, via direct effect of TNF-*α* on insulin signaling [35, 36]. Patients with diabetes demonstrate high expression of TNF-*α* in skeletal muscle and in plasma, and it is likely that adipose tissue, which produces TNF-*α*, is the main source of the circulating TNF-*α* [37–39].

Thus, a number of studies indicate that elevated TNF-*α* is not secondary to the pathological conditions associated with insulin resistance, but that TNF-*α* plays a direct pathogenic role in glucose metabolism [29, 40]. After adjustment for multiple confounders, including IL-6, high plasma TNF concentrations are associated with insulin resistance [40].

In cultured cells, TNF-*α* induces insulin resistance through increased serine phosphorylationof insulin receptor substrate-1 (IRS-1), which subsequently converts IRS-1 to an inhibitor of insulin receptor tyrosinekinase activity [41].

TNF-*α* has been shown to have a direct (insulin-independent) effecton S6K and ERK-1/2 in cultured cells [42, 43].

In addition, evidence for a direct role of TNF-*α* in insulin resistance in humans in vivo has been obtained [44]. When TNF-*α* was infused into healthy humans, we found that TNF-*α* induces insulin resistance in skeletal muscle, without an effect on endogenous glucose production. TNF-*α* directly impaired glucose uptake and metabolism by altering insulin signal transduction. TNF-alpha infusion increased phosphorylation of p70 S6 kinase, extracellular signal-regulated kinase-1/2, and c-Jun NH(2)-terminal kinase, concomitant with increased serine and reduced tyrosine phosphorylation of insulin receptor substrate-1. These signalling effects were associated with impaired phosphorylation of Akt substrate 160, the most proximal step identified in the canonical insulin signalling cascade regulating GLUT4 translocation and glucose uptake. Thus, excessive concentrations of TNF-alpha negatively regulate insulin signalling and whole-body glucose uptake in humans.

Moreover, it was recently demonstrated that TNF-alpha infusion increases whole-body lipolysis by 40% with a concomitant increase in FFA clearance, but with no changes in skeletal muscle FFA uptake, release, or oxidation [45].

The findings with regard to an effect of TNF on both glucose and fat metabolism provide a direct molecular link between low-grade systemic inflammation and the metabolic syndrome [44, 45].

## 4. THE DIRECT METABOLIC ROLES OF IL-6

With regard to IL-6, its role in insulin resistance is highly controversial. In humans, circulating IL-6 levels may [46] or may not [47] be associated with insulin resistance.

Infusion of recombinant human (rh) IL-6 into resting healthy humans does not impair whole body, lower limb, or subcutaneous adipose tissue glucose uptake or endogenous glucose production [48, 49]. When diabetes patients were given an rhIL-6 infusion, plasma concentrations of insulin decreased to levels comparable with that in age and BMI (body mass index)-matched healthy controls, indicating that the IL-6 enhanced insulin sensitivity [50].

In vitro studies demonstrate that IL-6 can induce insulin resistance in isolated 3T3-L1 adipocytes [51, 52] and in mice [53]. However, the IL-6 dose applied in the latter studies was supraphysiological, and is therefore probably not relevant to human physiology. Interestingly, IL-6 knockout mice develop impaired glucose tolerance that is reverted by IL-6. Thus, accumulating data suggest that IL-6 enhances glucose uptake in myocytes [54–57].

A number of studies indicate that IL-6 enhances lipolysis, as well as fat oxidation [50, 58]. Consistent with this idea, Wallenius et al. 2002 demonstrated that IL-6 deficient mice developed mature-onset obesity and insulin resistance. In addition, when the mice were treated with IL-6, there was a significant decrease in body fat mass in the IL-6 knockout but not in the wild-type mice. Recently, we demonstrated that IL-6 increased the glucose infusion rate [56] and glucose oxidation without affecting the suppression of endogenous glucose production during a hyperinsulinemic euglycemic clamp in healthy humans. Infusion of rhIL-6 into healthy humans to obtain physiological concentrations of IL-6 increased lipolysis in the absence of hypertriacylglyceridemia or changes in catecholamines, glucagon, insulin, or any adverse effects in healthy individuals as well as patients with type 2 diabetes [49, 50, 58]. These findings, together with cell culture experiments demonstrating that IL-6 alone markedly increases both lipolysis and fat oxidation, identify IL-6 as a novel lipolytic factor.

Taken together, it is apparent that in vivo studies in humans provide little or no evidence that IL-6 is a direct player in the metabolic syndrome.

## 5. TNF/IL-6-INTERACTION

Of note, whereas it is known that both TNF-*α* and IL-6 induce lipolysis [40, 49], only IL-6 appears to induce fat oxidation [19, 59]. Given the different biological profiles of TNF-*α* and IL-6, and given that TNF-*α* can trigger IL-6 release, one theory holds that it is the adipose tissue-derived TNF-*α* that actually is the “driver” behind the metabolic syndrome [36] and that locally produced TNF-*α* causes increased systemic levels of IL-6.

## 6. CYTOKINE RESPONSES TO EXERCISE

Though an acute bout of physical activity is accompanied by responses that in many respects are similar to those induced by infection and sepsis, there are some important differences in the cytokine response to exercise from that elicited by severe infection [27, 60, 61]. The striking difference between exercise and sepsis with regard to cytokine responses is that the classical proinflammatory cytokines, TNF-*α* and IL-1*β*, in general do not increase with exercise.

Typically, IL-6 is the first cytokine present in the circulation during exercise, and the appearance of IL-6 in the circulation is by far the most marked and its appearance precedes that of the other cytokines. The level of circulating IL-6 increases in an exponential fashion (up to 100 fold) in response to exercise, and declines in the postexercise period [60–62].

Taken together, exercise provokes an increase primarily in IL-6, followed by an increase in IL-1ra and IL-10. The IL-6 response to exercise has recently been reviewed [13, 18, 60, 63]. A marked increase in circulating levels of IL-6 after exercise without muscle damage has been a remarkably consistent finding. The magnitude by which plasma IL-6 increases is related to exercise duration, intensity, and muscle mass involved in the mechanical work [13, 18, 60, 62].

## 7. ANTI-INFLAMMATORY EFFECTS OF IL-6

Interleukin-6 is most often classified as a proinflammatory cytokine, although data also suggest that IL-6 and IL-6-regulated acute phase proteins are anti-inflammatory and immunosuppressive, and may negatively regulate the acute-phase response [20].

A number of studies have demonstrated that working muscle produces IL-6. Thus muscle biopsies obtained before and after exercise in humans and rats demonstrate very little IL-6 mRNA in resting muscle, but up to a 100-fold increase in exercising skeletal muscle [18, 64, 65]. In addition, it has been demonstrated that the IL-6 protein is expressed in contracting muscle fibres and that IL-6 is released from skeletal muscle during exercise [28]. Data suggest that IL-6 exerts inhibitory effects on TNF-*α* and IL-1 production. IL-6 inhibits lipopolysaccharide (LPS)-induced TNF-*α* production both in cultured human monocytes and in the human monocytic line U937 [66], and levels of TNF-*α* are markedly elevated in anti-IL-6-treated mice and in IL-6-deficient knockout mice [67, 68], indicating that circulating IL-6 is involved in the regulation of TNF-*α* levels. In addition, rhIL-6 infusion inhibits the endotoxin-induced increase in circulating levels of TNF-*α* in healthy humans [69]. Furthermore, IL-6 stimulates the release of soluble TNF-*α* receptors, but not IL-1*β* and TNF-*α*, and appears to be the primary inducer of the hepatocyte-derived acute-phase proteins, many of which have anti-inflammatory properties.

The exercise-induced increase in plasma IL-6 levels is followed by increased circulating levels of well-known anti-inflammatory cytokines such as IL-1ra and IL-10 [28, 48].

It has been suggested that IL-6 promotes insulin resistance due to the observation that plasma IL-6 is often elevated in patients with metabolic disease [70].

From a simplistic physiological point of view, it seems paradoxical that working muscle would release a factor that inhibits insulin signalling when insulin sensitivity is enhanced in the immediate postexercise period [28].

## 8. ANTI-INFLAMMATORY EFFECTS OF EXERCISE

A number of studies suggest that regular exercise has anti-inflammatory effects. Cross-sectional studies demonstrate an association between physical inactivity and low-grade systemic inflammation in healthy subjects [71–75], in elderly people [76, 77], and in patients with intermittent claudication [78]. Moreover, the finding in longitudinal studies that regular training induces a reduction in CRP level suggests that the physical activity as such may suppress systemic low-grade inflammation [74, 75, 79, 80].

To study whether acute exercise induces a true anti-inflammatory response, a model of “low-grade inflammation" was established in which we injected a low dose of *Escherichia coli* endotoxin to healthy volunteers, who had been randomized to either rest or exercise prior to endotoxin administration. In resting subjects, endotoxin induced a 2- to 3-fold increase in circulating levels of TNF-*α*. In contrast, when the subjects performed 3 hours of ergometer cycling and received the endotoxin bolus at 2.5 hours, the TNF-*α* response was totally blunted. Moreover, the effect of exercise could be mimicked by infusion of IL-6, suggesting that IL-6 may be involved in mediating the anti-inflammatory effects of exercise [69].

## 9. CONCLUSION

Regular exercise protects against diseases associated with chronic low-grade systemic inflammation. Muscle contraction-induced factors, so-called myokines, may be involved in mediating the health beneficial effects of exercise and play important roles in the protection against CNCDs, which include cardiovascular conditions, some cancers, and type 2 diabetes. In particular, the long-term effect of exercise may to some extent be ascribed to the anti-inflammatory response elicited by an acute bout of exercise, which is partly mediated by muscle-derived IL-6 [8, 20, 81]. The possibility exists that, with regular exercise, the anti-inflammatory effects of an acute bout of exercise will protect against chronicsystemic low-grade inflammation and thereby offer protection against insulin resistance and the development of atherosclerosis, but such a link between the acute effects of exercise and the long-term benefits has not yet been proven. Given that the atherosclerotic process is characterized by inflammation, one alternative explanation would be that regular exercise, which offers protection against atherosclerosis, indirectly offers protection against vascular inflammation and hence systemic low-grade inflammation.
